# Supplementary material for: Mapping the evolution of fertility support policies in China: A content and instrumental analysis
Source: PLoS One. 2025 Oct 9;20(10):e0332137. doi: 10.1371/journal.pone.0332137 (PMC12510515; doi:10.1371/journal.pone.0332137)
Supplement: S1 Appendix — (ZIP) [file pone.0332137.s001.zip › S1 Appendix. 226 original policy documents/139-中共中央、国务院关于支持浙江高质量发展建设共同富裕示范区的意见(FBM-CLI-16-5015164).doc]

中共中央、国务院关于支持浙江高质量发展建设共同富裕示范区的意见

**发布部门：** [中国共产党中央委员会](https://www.pkulaw.com/law/chl?Aggs.IssueDepartment=a01) [国务院](https://www.pkulaw.com/law/chl?Aggs.IssueDepartment=5)

**发布日期：**2021.05.20

**实施日期：**2021.05.20

**时效性：** [现行有效](https://www.pkulaw.com/law/chl?Aggs.TimelinessDic=01)

**效力级别：** [党内法规制度](https://www.pkulaw.com/law/chl?Aggs.EffectivenessDic=XR12)

**法规类别：** [区域经济开发](https://www.pkulaw.com/law/chl?Aggs.Category=02308) [营商环境优化](https://dobusiness.pkulaw.com/)

**专题分类：** [区域](https://www.pkulaw.com/law/chl?Aggs.SpecialType=003012)

中共中央、国务院关于支持浙江高质量发展建设共同富裕示范区的意见
（2021年5月20日）

　　共同富裕是社会主义的本质要求，是人民群众的共同期盼。改革开放以来，通过允许一部分人、一部分地区先富起来，先富带后富，极大解放和发展了社会生产力，人民生活水平不断提高。党的十八大以来，以习近平同志为核心的党中央不忘初心、牢记使命，团结带领全党全国各族人民，始终朝着实现共同富裕的目标不懈努力，全面建成小康社会取得伟大历史性成就，特别是决战脱贫攻坚取得全面胜利，困扰中华民族几千年的绝对贫困问题得到历史性解决，为新发展阶段推动共同富裕奠定了坚实基础。
　　党的十九届五中全会对扎实推动共同富裕作出重大战略部署。实现共同富裕不仅是经济问题，而且是关系党的执政基础的重大政治问题。共同富裕具有鲜明的时代特征和中国特色，是全体人民通过辛勤劳动和相互帮助，普遍达到生活富裕富足、精神自信自强、环境宜居宜业、社会和谐和睦、公共服务普及普惠，实现人的全面发展和社会全面进步，共享改革发展成果和幸福美好生活。随着我国开启全面建设社会主义现代化国家新征程，必须把促进全体人民共同富裕摆在更加重要的位置，向着这个目标更加积极有为地进行努力，让人民群众真真切切感受到共同富裕看得见、摸得着、真实可感。
　　当前，我国发展不平衡不充分问题仍然突出，城乡区域发展和收入分配差距较大，各地区推动共同富裕的基础和条件不尽相同。促进全体人民共同富裕是一项长期艰巨的任务，需要选取部分地区先行先试、作出示范。浙江省在探索解决发展不平衡不充分问题方面取得了明显成效，具备开展共同富裕示范区建设的基础和优势，也存在一些短板弱项，具有广阔的优化空间和发展潜力。支持浙江高质量发展建设共同富裕示范区，有利于通过实践进一步丰富共同富裕的思想内涵，有利于探索破解新时代社会主要矛盾的有效途径，有利于为全国推动共同富裕提供省域范例，有利于打造新时代全面展示中国特色社会主义制度优越性的重要窗口。现就支持浙江高质量发展建设共同富裕示范区提出如下意见。
　　一、总体要求
　　（一）指导思想。以习近平新时代中国特色社会主义思想为指导，深入贯彻党的十九大和十九届二中、三中、四中、五中全会精神，全面贯彻落实习近平总书记关于浙江工作的重要指示批示精神，坚持稳中求进工作总基调，坚持以人民为中心的发展思想，立足新发展阶段、贯彻新发展理念、构建新发展格局，紧扣推动共同富裕和促进人的全面发展，坚持以满足人民日益增长的美好生活需要为根本目的，以改革创新为根本动力，以解决地区差距、城乡差距、收入差距问题为主攻方向，更加注重向农村、基层、相对欠发达地区倾斜，向困难群众倾斜，支持浙江创造性贯彻“八八战略”，在高质量发展中扎实推动共同富裕，着力在完善收入分配制度、统筹城乡区域发展、发展社会主义先进文化、促进人与自然和谐共生、创新社会治理等方面先行示范，构建推动共同富裕的体制机制，着力激发人民群众积极性、主动性、创造性，促进社会公平，增进民生福祉，不断增强人民群众的获得感、幸福感、安全感和认同感，为实现共同富裕提供浙江示范。

　　（二）工作原则
　　--坚持党的全面领导。坚定维护党中央权威和集中统一领导，充分发挥党总揽全局、协调各方的领导核心作用，坚持和完善中国特色社会主义制度，把党的政治优势和制度优势转化为推动共同富裕示范区建设、广泛凝聚各方共识的强大动力和坚强保障。
　　--坚持以人民为中心。坚持发展为了人民、发展依靠人民、发展成果由人民共享，始终把人民对美好生活的向往作为推动共同富裕的奋斗目标，瞄准人民群众所忧所急所盼，在更高水平上实现幼有所育、学有所教、劳有所得、病有所医、老有所养、住有所居、弱有所扶。
　　--坚持共建共享。弘扬勤劳致富精神，鼓励劳动者通过诚实劳动、辛勤劳动、创新创业实现增收致富，不断提高劳动生产率和全要素生产率。充分发挥市场在资源配置中的决定性作用，更好发挥政府作用，体现效率、促进公平，坚决防止两极分化，在发展中补齐民生短板，让发展成果更多更公平惠及人民群众。
　　--坚持改革创新。坚定不移推进改革，推动有利于共同富裕的体制机制不断取得新突破，着力破除制约高质量发展高品质生活的体制机制障碍，强化有利于调动全社会积极性的重大改革开放举措。坚持创新在现代化建设全局中的核心地位，深入实施创新驱动发展战略，率先在推动共同富裕方面实现理论创新、实践创新、制度创新、文化创新。
　　--坚持系统观念。立足当前、着眼长远，统筹考虑需要和可能，按照经济社会发展规律循序渐进，脚踏实地、久久为功，不吊高胃口、不搞“过头事”，尽力而为、量力而行，注重防范化解重大风险，使示范区建设与经济发展阶段相适应、与现代化建设进程相协调，不断形成推动共同富裕的阶段性标志性成果。

　　（三）战略定位
　　--高质量发展高品质生活先行区。率先探索实现高质量发展的有效路径，促进城乡居民收入增长与经济增长更加协调，构建产业升级与消费升级协调共进、经济结构与社会结构优化互促的良性循环，更好满足人民群众品质化多样化的生活需求，富民惠民安民走在全国前列。
　　--城乡区域协调发展引领区。坚持城乡融合、陆海统筹、山海互济，形成主体功能明显、优势互补、高质量发展的国土空间开发保护新格局，健全城乡一体、区域协调发展体制机制，加快基本公共服务均等化，率先探索实现城乡区域协调发展的路径。
　　--收入分配制度改革试验区。坚持按劳分配为主体、多种分配方式并存，着重保护劳动所得，完善要素参与分配政策制度，在不断提高城乡居民收入水平的同时，缩小收入分配差距，率先在优化收入分配格局上取得积极进展。
　　--文明和谐美丽家园展示区。加强精神文明建设，推动生态文明建设先行示范，打造以社会主义核心价值观为引领、传承中华优秀文化、体现时代精神、具有江南特色的文化强省，实现国民素质和社会文明程度明显提高、团结互助友爱蔚然成风、经济社会发展全面绿色转型，成为人民精神生活丰富、社会文明进步、人与自然和谐共生的幸福美好家园。

　　（四）发展目标
　　到2025年，浙江省推动高质量发展建设共同富裕示范区取得明显实质性进展。经济发展质量效益明显提高，人均地区生产总值达到中等发达经济体水平，基本公共服务实现均等化；城乡区域发展差距、城乡居民收入和生活水平差距持续缩小，低收入群体增收能力和社会福利水平明显提升，以中等收入群体为主体的橄榄型社会结构基本形成，全省居民生活品质迈上新台阶；国民素质和社会文明程度达到新高度，美丽浙江建设取得新成效，治理能力明显提升，人民生活更加美好；推动共同富裕的体制机制和政策框架基本建立，形成一批可复制可推广的成功经验。
　　到2035年，浙江省高质量发展取得更大成就，基本实现共同富裕。人均地区生产总值和城乡居民收入争取达到发达经济体水平，城乡区域协调发展程度更高，收入和财富分配格局更加优化，法治浙江、平安浙江建设达到更高水平，治理体系和治理能力现代化水平明显提高，物质文明、政治文明、精神文明、社会文明、生态文明全面提升，共同富裕的制度体系更加完善。
　　二、提高发展质量效益，夯实共同富裕的物质基础

　　（五）大力提升自主创新能力。以创新型省份建设为抓手，把科技自立自强作为战略支撑，加快探索社会主义市场经济条件下新型举国体制开展科技创新的浙江路径。实施好关键核心技术攻关工程，强化国家战略科技力量，为率先实现共同富裕提供强劲内生动力。支持布局重大科技基础设施和平台，建设创新策源地，打造“互联网＋”、生命健康、新材料科创高地。高水平建设杭州、宁波温州国家自主创新示范区，深化国家数字经济创新发展试验区建设，强化“云上浙江”和数字强省基础支撑，探索消除数字鸿沟的有效路径，保障不同群体更好共享数字红利。畅通创新要素向企业集聚通道，鼓励企业组建创新联合体和知识产权联盟，建设共性技术平台。加大对科技成果应用和产业化的政策支持力度，打造辐射全国、链接全球的技术交易平台。

　　（六）塑造产业竞争新优势。巩固壮大实体经济根基，夯实共同富裕的产业基础。加快推进产业转型升级，大力推动企业设备更新和技术改造，推动传统产业高端化、智能化、绿色化发展，做优做强战略性新兴产业和未来产业，培育若干世界级先进制造业集群，打响“浙江制造”品牌。促进中小微企业专精特新发展，提升创新能力和专业化水平。推动农村一二三产业融合发展，建设农业现代化示范区，做精农业特色优势产业和都市农业，发展智慧农业。加快服务业数字化、标准化、品牌化发展，推动现代服务业同先进制造业、现代农业深度融合。畅通金融服务实体经济渠道。

　　（七）提升经济循环效率。落实构建新发展格局要求，贯通生产、分配、流通、消费各环节，在率先实现共同富裕进程中畅通经济良性循环。深化供给侧结构性改革，扩大优质产品和服务消费供给，加快线上线下消费双向深度融合。支持适销对路的优质外贸产品拓宽内销渠道。加快构建现代流通体系，推动海港、陆港、空港、信息港“四港”联动。统筹推进浙江自由贸易试验区各片区联动发展，开展首创性和差别化改革探索。畅通城乡区域经济循环，破除制约城乡区域要素平等交换、双向流动的体制机制障碍，促进城乡一体化、区域协调发展。支持浙江发挥好各地区比较优势，加强大湾区大花园大通道大都市区建设。更加主动对接上海、江苏、安徽，更好融入长三角一体化发展。加快建设“一带一路”重要枢纽，大力发展数字贸易、服务贸易，发展更高水平开放型经济。

　　（八）激发各类市场主体活力。推动有效市场和有为政府更好结合，培育更加活跃更有创造力的市场主体，壮大共同富裕根基。高水平推动浙江杭州区域性国资国企综合改革试验，完善国有资产监管体制，规范有序开展混合所有制改革，做强做优做大国有资本和国有企业，充分发挥国有经济战略支撑作用。完善产权保护制度，构建亲清政商关系，促进非公有制经济健康发展和非公有制经济人士健康成长，破除制约民营企业发展的各种壁垒，完善促进中小微企业和个体工商户发展的法律环境和政策体系，建立企业减负长效机制。加快建设高标准市场体系，持续优化市场化法治化国际化营商环境，实施统一的[市场准入负面清单](https://www.pkulaw.com/chl/0034c9a92059f448bdfb.html)制度。坚持发展和规范并重，建立健全平台经济治理体系，督促平台企业承担质量和安全保障等责任，推动平台经济为高质量发展和高品质生活服务。加大反垄断和反不正当竞争执法司法力度，提升监管能力和水平，实现事前事中事后全链条监管，防止资本无序扩张。
　　三、深化收入分配制度改革，多渠道增加城乡居民收入

　　（九）推动实现更加充分更高质量就业。强化就业优先政策，坚持经济发展就业导向，扩大就业容量，提升就业质量，促进充分就业。支持和规范发展新就业形态，完善促进创业带动就业、多渠道灵活就业的保障制度。统筹各类职业技能培训资金，合理安排就业补助资金，健全统筹城乡的就业公共服务体系。鼓励返乡入乡创业。完善重点群体就业支持体系，帮扶困难人员就业。创造公平就业环境，率先消除户籍、地域、身份、性别等影响就业的制度障碍，深化构建和谐劳动关系，推动劳动者通过辛勤劳动提高生活品质。

　　（十）不断提高人民收入水平。优化政府、企业、居民之间分配格局，支持企业通过提质增效拓展从业人员增收空间，合理提高劳动报酬及其在初次分配中的比重。健全工资合理增长机制，完善企业薪酬调查和信息发布制度，合理调整最低工资标准，落实带薪休假制度。完善创新要素参与分配机制，支持浙江加快探索知识、技术、管理、数据等要素价值的实现形式。拓宽城乡居民财产性收入渠道，探索通过土地、资本等要素使用权、收益权增加中低收入群体要素收入。丰富居民可投资金融产品，完善上市公司分红制度。鼓励企业开展员工持股计划。深入推进农村集体产权制度改革，巩固提升农村集体经济，探索股权流转、抵押和跨社参股等农村集体资产股份权能实现新形式。立足当地特色资源推动乡村产业发展壮大，完善利益联结机制，让农民更多分享产业增值收益。支持浙江率先建立集体经营性建设用地入市增值收益分配机制。

　　（十一）扩大中等收入群体。实施扩大中等收入群体行动计划，激发技能人才、科研人员、小微创业者、高素质农民等重点群体活力。加大人力资本投入力度，健全面向劳动者的终身职业技能培训制度，实施新时代浙江工匠培育工程，加快构建产教训融合、政企社协同、育选用贯通的技术技能人才培养培训体系，完善技能人才薪酬分配政策，拓宽技术工人上升通道。对有劳动能力的低收入群体，坚持开发式帮扶，提高内生发展能力，着力发展产业使其积极参与就业。拓展基层发展空间，保障不同群体发展机会公平，推动更多低收入群体迈入中等收入群体行列。规范招考选拔聘用制度，完善评价激励机制。完善党政机关、企事业单位和社会各方面人才顺畅流动的制度体系。实行更加开放的人才政策，激发人才创新活力。

　　（十二）完善再分配制度。支持浙江在调节收入分配上主动作为，加大省对市县转移支付等调节力度和精准性，合理调节过高收入。依法严厉惩治贪污腐败，继续遏制以权力、行政垄断等非市场因素获取收入，取缔非法收入。优化财政支出结构，加大保障和改善民生力度，建立健全改善城乡低收入群体等困难人员生活的政策体系和长效机制。

　　（十三）建立健全回报社会的激励机制。鼓励引导高收入群体和企业家向上向善、关爱社会，增强社会责任意识，积极参与和兴办社会公益事业。充分发挥第三次分配作用，发展慈善事业，完善有利于慈善组织持续健康发展的体制机制，畅通社会各方面参与慈善和社会救助的渠道。探索各类新型捐赠方式，鼓励设立慈善信托。加强对慈善组织和活动的监督管理，提高公信力和透明度。落实公益性捐赠税收优惠政策，完善慈善褒奖制度。
　　四、缩小城乡区域发展差距，实现公共服务优质共享

　　（十四）率先实现基本公共服务均等化。推进城乡区域基本公共服务更加普惠均等可及，稳步提高保障标准和服务水平。推动义务教育优质均衡发展，建成覆盖城乡的学前教育公共服务体系，探索建立覆盖全省中小学的新时代城乡教育共同体，共享“互联网＋教育”优质内容，探索终身学习型社会的浙江示范，提高人口平均受教育年限和综合能力素质。深入实施健康浙江行动，加快建设强大的公共卫生体系，深化县域医共体和城市医联体建设，推动优质医疗资源均衡布局。积极应对人口老龄化，提高优生优育服务水平，大力发展普惠托育服务体系，加快建设居家社区机构相协调、医养康养相结合的养老服务体系，发展普惠型养老服务和互助性养老。健全全民健身公共服务体系。

　　（十五）率先实现城乡一体化发展。高质量创建乡村振兴示范省，推动新型城镇化与乡村振兴全面对接，深入探索破解城乡二元结构、缩小城乡差距、健全城乡融合发展的体制机制。推动实现城乡交通、供水、电网、通信、燃气等基础设施同规同网。推进以人为核心的新型城镇化，健全农业转移人口市民化长效机制，探索建立人地钱挂钩、以人定地、钱随人走制度，切实保障农民工随迁子女平等接受义务教育，逐步实现随迁子女入学待遇同城化。促进大中小城市与小城镇协调发展。推进以县城为重要载体的城镇化建设，推进空间布局、产业发展、基础设施等县域统筹，赋予县级更多资源整合使用的自主权。以深化“千村示范、万村整治”工程牵引新时代乡村建设。

　　（十六）持续改善城乡居民居住条件。坚持房子是用来住的、不是用来炒的定位，完善住房市场体系和住房保障体系，确保实现人民群众住有所居。针对新市民、低收入困难群众等重点群体，有效增加保障性住房供给。对房价比较高、流动人口多的城市，土地供应向租赁住房建设倾斜，探索利用集体建设用地和企事业单位自有闲置土地建设租赁住房，扩大保障性租赁住房供给，加快完善长租房政策，使租购住房在享受公共服务上具有同等权利。全面推进城镇老旧小区改造和社区建设，提升农房建设质量，加强农村危房改造，探索建立农村低收入人口基本住房安全保障机制，塑造江南韵、古镇味、现代风的新江南水乡风貌，提升城乡宜居水平。

　　（十七）织密扎牢社会保障网。完善社会保障制度，加快实现法定人员全覆盖，建立统一的社保公共服务平台，实现社保事项便捷“一网通办”。健全多层次、多支柱养老保险体系，大力发展企业年金、职业年金、个人储蓄型养老保险和商业养老保险。规范执行全国统一的社保费率标准。推动基本医疗保险、失业保险、工伤保险省级统筹。健全重大疾病医疗保险制度。做好长期护理保险制度试点工作，积极发展商业医疗保险。健全灵活就业人员社保制度。健全统一的城乡低收入群体精准识别机制，完善分层分类、城乡统筹的社会救助体系，加强城乡居民社会保险与社会救助制度的衔接，按困难类型分类分档及时给予专项救助、临时救助，切实兜住因病、因灾致贫等困难群众基本生活底线。保障妇女儿童合法权益，完善帮扶残疾人、孤儿等社会福利制度。

　　（十八）完善先富带后富的帮扶机制。加快推进省以下财政事权和支出责任划分改革，加大向重点生态功能区的转移支付力度。强化陆海统筹，升级山海协作工程，挖掘海域和山区两翼的潜力优势，支持一批重点生态功能区县增强内生发展能力和实力，带动山区群众增收致富。全域参与海洋经济发展，建设海洋强省。探索建立先富帮后富、推动共同富裕的目标体系、工作体系、政策体系、评估体系。深入实施东西部协作和对口支援，持续推进智力支援、产业支援、民生改善、文化教育支援，加强对省外欠发达地区帮扶，大力推进产业合作、消费帮扶和劳务协作，探索共建园区、飞地经济等利益共享模式。完善社会力量参与帮扶的长效机制。
　　五、打造新时代文化高地，丰富人民精神文化生活

　　（十九）提高社会文明程度。推动学习贯彻习近平新时代中国特色社会主义思想走深走心走实，实现理想信念教育常态化制度化。坚持以社会主义核心价值观为引领，加强爱国主义、集体主义、社会主义教育，厚植勤劳致富、共同富裕的文化氛围。推进公民道德建设，支持培育“最美浙江人”等品牌。扎实推进新时代文明实践中心建设，深入实施文明创建工程，打造精神文明高地。完善覆盖全省的现代公共文化服务体系，提高城乡基本公共文化服务均等化水平，深入创新实施文化惠民工程，优化基层公共文化服务网络。弘扬诚信文化，推进诚信建设，营造人与人之间互帮互助、和睦友好的社会风尚。加强家庭家教家风建设，健全志愿服务体系，广泛开展志愿服务关爱行动。

　　（二十）传承弘扬中华优秀传统文化、革命文化、社会主义先进文化。传承弘扬中华优秀传统文化，充分挖掘浙江文化优势，深入推进大运河国家文化公园、大运河文化带建设，振兴非遗记忆。传承红色基因，大力弘扬革命文化，提升爱国主义教育基地建设水平。实施重大文化设施建设工程，打造具有国际影响力的影视文化创新中心和数字文化产业集群，提供更多优秀文艺作品、优秀文化产品和优质旅游产品，更好满足人民群众文化需求。
　　六、践行绿水青山就是金山银山理念，打造美丽宜居的生活环境

　　（二十一）高水平建设美丽浙江。支持浙江开展国家生态文明试验区建设，绘好新时代“富春山居图”。强化国土空间规划和用途管控，优化省域空间布局，落实生态保护、基本农田、城镇开发等空间管控边界。坚持最严格的耕地保护制度和最严格的节约用地制度，严格规范执行耕地占补平衡制度，对违法占用耕地“零容忍”，坚决有效遏制增量，依法有序整治存量，强化耕地数量保护和质量提升。深化生态文明体制改革，实行最严格的生态环境保护制度，健全明晰高效的自然资源资产产权制度。坚持山水林田湖草系统治理，全面提升生物多样性保护水平。完善生态保护补偿机制，推广新安江等跨流域共治共保共享经验。继续打好蓝天、碧水、净土保卫战，强化多污染物协同控制和区域协同治理，推进生态环境持续改善。推进海岸带综合保护与利用。推进海岛特色化差异化发展，加强海岛生态环境保护。

　　（二十二）全面推进生产生活方式绿色转型。拓宽绿水青山就是金山银山转化通道，建立健全生态产品价值实现机制，探索完善具有浙江特点的生态系统生产总值（GEP）核算应用体系。高标准制定实施浙江省碳排放达峰行动方案。推进排污权、用能权、用水权市场化交易，积极参与全国碳排放权交易市场。大力发展绿色金融。全面促进能源资源节约集约利用，进一步推进生活垃圾分类，加快构建家电、汽车等废旧物资循环利用体系。深化“无废城市”建设。大力推行简约适度、绿色低碳、文明健康的生活方式，广泛开展绿色生活创建行动，促进人与自然和谐共生。
　　七、坚持和发展新时代“枫桥经验”，构建舒心安心放心的社会环境

　　（二十三）以数字化改革提升治理效能。强化数字赋能，聚焦党政机关整体智治、数字经济、数字社会、数字政府、数字法治等领域，探索智慧治理新平台、新机制、新模式。推进“互联网＋放管服”，全面推行“掌上办事”、“掌上办公”。深化“一件事”集成改革。健全党组织领导的自治、法治、德治、智治融合的城乡基层治理体系，完善基层民主协商制度，推进市域社会治理现代化，建设人人有责、人人尽责、人人享有的社会治理共同体。推进“最多跑一地”改革，完善县级社会矛盾纠纷调处化解中心工作机制。

　　（二十四）全面建设法治浙江、平安浙江。健全覆盖城乡的公共法律服务体系，加大普法力度，推动尊法学法守法用法，促进公平正义，建设法治社会。构建全覆盖的政府监管体系和行政执法体系。高水平建设平安中国示范区，把保护人民生命安全摆在首位，加强社会治安防控体系建设，全面提高公共安全保障能力。建立健全覆盖各领域各方面的风险监测防控平台，健全防范化解重大风险挑战体制机制，守住不发生系统性风险底线。
　　八、保障措施

　　（二十五）坚持和加强党的全面领导。把党的领导贯穿推动浙江高质量发展建设共同富裕示范区的全过程、各领域、各环节。落实全面从严治党主体责任、监督责任，持之以恒加强党风廉政建设，不断深化清廉浙江建设，营造风清气正的良好政治生态。以正确用人导向引领干部干事创业，落实“三个区分开来”要求，做好容错纠错工作，加强对敢担当善作为干部的激励保护。

　　（二十六）强化政策保障和改革授权。中央和国家机关有关部门要结合自身职能，加强对浙江省的指导督促，根据本意见有针对性制定出台专项政策，优先将本领域改革试点、探索示范任务赋予浙江，并加强对改革试验、政策实施的监督检查。根据浙江高质量发展建设共同富裕示范区需要，在科技创新、数字化改革、分配制度改革、城乡区域协调发展、公共服务、生态产品价值实现等方面给予改革授权。涉及重要政策、重要规划、重大项目的，要依法依规办理并按程序报批。有关改革政策措施凡涉及调整现行法律或行政法规的，按法定程序经全国人大常委会或国务院统一授权后实施。

　　（二十七）建立评价体系和示范推广机制。加快构建推动共同富裕的综合评价体系，建立评估机制，坚持定量与定性、客观评价与主观评价相结合，全面反映共同富裕示范区建设工作成效，更好反映人民群众满意度和认同感。建立健全示范推广机制，及时总结示范区建设的好经验好做法，归纳提炼体制机制创新成果，成熟一批、推广一批，发挥好对全国其他地区的示范带动作用。

　　（二十八）完善实施机制。健全中央统筹、省负总责、市县抓落实的实施机制。依托推动长三角一体化发展领导小组，加强对浙江建设共同富裕示范区的统筹指导，国家发展改革委牵头设立工作专班负责协调推进本意见提出的任务措施。浙江省要切实承担主体责任，增强敢闯敢试、改革破难的担当精神，始终保持奋进姿态，立足省情和发展实际，制定具体实施方案，充分动员各方力量，不断开辟干在实处、走在前列、勇立潮头新境界。重大事项及时向党中央、国务院请示报告。

（新华社北京2021年6月10日电）

© 北大法宝：（[www.pkulaw.com](https://www.pkulaw.com/)) 专业提供法律信息、法学知识和法律软件领域各类解决方案。北大法宝为您提供丰富的参考资料，正式引用法规条文时请与标准文本核对。
欢迎查看所有[产品和服务](https://www.pkulaw.com/helps/2.html)。法宝快讯：[如何快速找到您需要的检索结果？](https://www.pkulaw.com/helps/70.html)[法宝](https://www.pkulaw.com/helps/69.html)V6有何新特色？

扫描二维码阅读原文

**原文链接：**<https://www.pkulaw.com/chl/232b17465a5cda4cbdfb.html>
